# End-of-life care in rural general practice: how best to support commitment and meet challenges?

**DOI:** 10.1186/s12904-019-0435-4

**Published:** 2019-06-25

**Authors:** Jinfeng Ding, Christobel Saunders, Angus Cook, Claire E. Johnson

**Affiliations:** 1 School of Population and Global Health, The University of Western Australia, 35 Stirling Highway, Perth, Western Australia 6009 Australia; 2 Medical School, The University of Western Australia, 35 Stirling Highway, Perth, WA 6009 Australia; 3 School of Nursing and Midwifery, Monash University, Wellington Road, Clayton, Victoria 3800 Australia

**Keywords:** End-of-life care, Palliative care, General practitioners, Other stakeholders, Qualitative study

## Abstract

**Background:**

Few studies have specifically assessed the scope, nature and challenges of palliative and end-of-life care in rural general practice. These knowledge gaps limit the development of evidence-based policies and services for patients in the last months of life. This study aimed to explore the perspectives of general practitioners (GPs) and other stakeholders on rural GPs’ involvement and challenges in providing palliative and end-of-life care in regional Australia.

**Methods:**

A qualitative study involving five focus groups with 26 GPs based in rural/regional Western Australia together with 15 individual telephone interviews with four GPs and 11 other stakeholders involved in end-of-life care across Australia.

**Results:**

The rural GPs’ central role in end-of-life care was recognized by the majority of participants but multiple challenges were also identified. Some challenges were comparable to those found in urban settings but others were more pronounced, including resource limitations and lack of training. Inappropriate payment models discouraged GPs’ involvement in some aspects of end-of-life care, such as case conferences and home visits. Compared to GPs in urban settings, those in rural/regional communities often reported closer doctor-patient relationships and better care integration and collaboration. These positive aspects of care could be further developed to enhance service provision. Our study highlighted the importance of regular interactions with other professionals and patients in providing end-of-life care, but many GPs and other stakeholders found such interactions more challenging than the more “technical” aspects of care.

**Conclusions:**

Rural/regional GPs appear to be disproportionately affected by inappropriate payment models and limited resources, but may benefit from closer doctor-patient relationships and better care integration and collaboration relative to urban GPs. Systematic collection of empirical data on GP management at end-of-life is required to build on these strengths and address the challenges.

**Electronic supplementary material:**

The online version of this article (10.1186/s12904-019-0435-4) contains supplementary material, which is available to authorized users.

## Background

There is an increasing gap between demand for palliative care in an ageing population and the supply of specialist palliative care services [1, 2]. Ensuring access to cost-effective community-based palliative and end-of-life (EOL) care has become a policy priority in most developed nations [3–5]. International studies have found gaps in palliative care availability and substantial challenges in providing this care in rural areas [6–9] and called for tailored palliative care strategies for rural settings [7, 8, 10, 11]. Although a third of Australians live outside major metropolitan centres [12], only 16% (36 out of 226) of palliative care specialists and 28% (796 out of 3457) of palliative care nurses worked in rural/regional areas based on a 2016 estimate [13].

In rural/regional Australia, general practitioners (GPs) are the principal local healthcare providers for patients with end-stage conditions [14, 15]. As clinical gatekeepers, they refer those patients with complex palliative care needs to clinical specialists or nurse-led palliative care teams. Based on recent surveys, rural Australian GPs were reportedly more committed to palliative and EOL care but received less specialist support than urban GPs [16, 17]. An interview-based study in 2006 noted that rural GPs in Western Australia (WA) perceived the provision of palliative care as a fundamental part of their role [18]. However, there are ongoing uncertainties around the scope and consistency of palliative and EOL care within general practice, including suboptimal symptom management [19–21], limited awareness of many patients’ preferred place of death [22] and low prevalence of advance care planning in general practice [23].

Few studies have specifically described rural/regional GPs’ involvement and challenges in providing palliative and EOL care [24–26]. The current knowledge gaps have limited policy responses that might better support GPs in the delivery of this care in rural/regional settings. This study aimed to achieve a comprehensive understanding of rural/regional GPs provision of palliative and EOL care in settings by exploring the perspectives of a wide range of stakeholders in relation to (i) the scope of palliative and EOL care provided by GPs in rural/regional Australia; and (ii) any challenges experienced by GPs in providing complete and consistent care in this context.

## Methods

### Study design

This study used a qualitative descriptive design involving face-to-face focus groups and telephone interviews. Qualitative description is widely adopted in studies [27] that seek firsthand knowledge on direct experiences from participants involved in the phenomenon of interest [28, 29]. We considered this design suitable for this study because we aimed to gain a diverse range of responses on the issue of rural GPs’ involvement and challenges in providing palliative and EOL care.

### Setting

The study was conducted in rural/regional areas in the Great Southern and South-west regions of WA (a combined area of 63,007 km^2^ and population of 237,000) [30, 31]. Each region has a main centre with a palliative medicine specialist, an inpatient hospice, a nurse-led community palliative care service with access to a social worker, and a generalist domiciliary nursing and home care service. The community services are available in the home as required by the patient and the patient’s family/carer, and are often dependent upon the progress of the disease and the availability and expertise of the carer. Access to the services of the palliative care team varies for communities outside of these regional centres, and generally decreases with distance. All GPs are able to use the state-wide 24-h palliative care specialist advice hotline.

### Participants and recruitment

This study comprised two groups of participants: (i) State registered medical practitioners with approved general practice qualifications who care for patients across all age groups and clinical conditions; (ii) Other stakeholders whose work was relevant to palliative and EOL care in rural/regional areas at the time of data collection, including selected policy makers, palliative care specialists and palliative care researchers from across Australia.

**Fig. 1:**
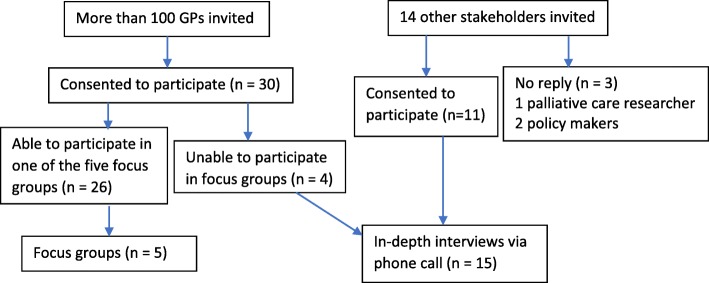
Flowchart for recruitment process

We recruited GPs using convenience sampling. Invitation emails were sent to GPs (*n* > 100) through local GP organizations, contacts of the researchers and the Western Australian Primary Health Alliance [32]. Purposive sampling was undertaken using researchers’ existing networks to identify 14 other stakeholders involved in community palliative and EOL care, and 11 of them agreed to participate. Figure 1 illustrates recruitment process.

### Data collection

Face-to-face focus groups with GPs were selected for data collection in order to encourage free exchange of information and to yield richer data and deeper insights into palliative and EOL care [33]. Five focus groups (average duration 46 min; range 36–57 min) involving 26 GPs (2–7 GPs in each group) took place at GPs’ offices and local community centres. Characteristics of these 26 GPs are presented in Table 1. Four GPs who were unable to participate in focus groups were invited to participate in in-depth telephone interviews. Given the heterogeneous characteristics of their work and their diverse locations around Australia, other 11 non-GP stakeholders also provided input through in-depth telephone interviews (average duration 34 min; range 18–55 min).

**Table 1:** Characteristics of GPs involved in focus groups

Characteristics	
Gender	
Male: 16 (61.5%)	
Female: 10 (38.5%)	
Age (years)	
Mean: 46.3	
Median: 45.5	
Range: 32–82	
Years of working as a GP	
Mean: 17.9	
Median: 15	
Range: 1–51	
Hours of working each week	
Mean: 38.4	
Median: 40	
Range 10–60	
Current work place	
Regional area: 7 (26.9%)	
Rural area: 18 (69.2%)	
Remote area: 1 (3.9%)	

Two researchers were present in each focus group. All researchers are health professionals (nurses, epidemiologist, and surgeon) with a research interest in palliative care and have qualitative research background. Authors C.J. and A.C. separately completed the in-depth interviews. The interview guides (see Additional file 1) were based on previous literature and consensus among the researchers, and were provided to participants 2 weeks before interviews. Guide questions focused on GPs’ involvement and challenges in caring for patients with life-limiting diseases in their last year of life. General practitioners were asked questions based on the care of their last two deceased patients. All focus groups and interviews were audio-taped and field notes were taken simultaneously.

### Analysis

Data collection was conducted between April and December 2017 until data saturation [34]. All recordings were transcribed verbatim. Thematic analysis was conducted using strategies described by Braun and Clarke (i.e. familiarizing with data; generating initial codes; searching for themes; reviewing themes; defining and naming themes; producing reports) with assistance of NVivo 11 [35]. J.D., who was trained as a nurse and researcher in China and has limited experience of Australian EOL care, first openly coded two focus groups and two interviews using an inductive approach. These preliminary coding results were shared with A.C. and C.J. for review. A discussion took place to achieve consensus on coding among the three authors. Afterwards, J.D. independently completed all the coding and thematic identification processes.

### Ethical considerations

This study received approval from Human Research Ethics Committee at the University of Western Australia with reference number RA/4/1/8895. We obtained both written and verbal consent from all participants. Recordings and transcriptions were stored in an encrypted computer and access only limited to researchers. All quotations are anonymized in this report.

## Results

Three broad categories of responses were identified: (i) Involvement in EOL care; (ii) Motivators for providing EOL care; (iii) Challenges/barriers in providing EOL care.

### Rural GPs’ involvement in EOL care

All participating GPs regarded providing EOL care as their responsibility and many agreed that their involvement in EOL care was welcomed by their patients. They were involved not only in the basic “clinical” aspects of palliative care (such as symptom control), but also in the management of the relationships with other care providers and care recipients (including negotiation on patients’ care with conflicting and estranged family members).

*I think the GP should communicate with the patients, … should arrange care arrangements, … should communicate with all of the facilities,* [and] *should have a good bond with the patients and the relatives and then organizing counselling, rather than only the medications.* [FG1-GP4].

Many GPs agreed that the interactions with patients and other care facilities was more challenging than the basic “practical” components of palliative care.

*One of the thoughts that I generated earlier was the actual medicine of palliative care is relatively straightforward. That’s not where the difficulty and complexity comes in. It’s the negotiation of everything else around. That’s where the real work* [is]*. In my experience, so that’s negotiating where does the right balance sit, where is the right direction to go, getting everyone to come to a reasonable agreement to have a good outcome …* [FG4-GP4].

Although rural GPs’ central role in providing EOL care was widely acknowledged by participants, one palliative care researcher challenged this, noting that EOL care was actually directed by specialists in most situations, with limited involvement from GPs.

*… I am not actually convinced that GPs are major providers for end of life care at all anymore. I think what happened last year, there were 160,000 deaths in Australia, 60, 000 are a bit more unexpected in one way or another. I think GPs were primary care providers for about 30,000 of the 160,000 …* [PCR3].

Some participants noted reduced GP engagement in EOL care in recent years, particularly in providing home visits and after-hours service. This corresponded with the increasing availability of community-based palliative care and locum services in rural/regional areas.

*At that time we* [GPs] *functioned as everything. There weren’t any palliative care specialists … But they* [palliative care specialists] *now look after them. I found, in fact, I am not so involved as much in their terminal care. They go to specialists and* [are] *referred by palliative care nurses, without necessarily our involvement …* [FG4-GP7].

Experienced GPs observed that the growth of specialisation may result in a perception among younger GPs that palliative care is difficult and beyond the scope of their practice. They suggested that more involvement in EOL care could lead to increased experience and confidence, and felt it was their responsibility to orientate and encourage younger GPs to engage with palliative care.

*The idea that you will only become involved if you had several years of training is not really correct. You need to encourage the younger doctors to get them involved. But obviously you need to back them up.* [ID-GP4].

Some GPs’ experience indicated that a higher level of involvement in patients’ care was required from GPs in more remote areas because of more limited access to specialist care.

*I* [GP] *was working in a smaller town where obviously I had greater role in patient’s care. The hospitals there were smaller and there were less specialists.* [ID-GP2].

### Motivators for providing EOL care

Many GPs reported that it was the emotional dimension of medicine that motivated their commitment to palliative and EOL care. In small towns, it was a common experience that patients and GPs have been “growing old together” and had built longstanding inter-personal relationships. A number of GPs viewed the patients’ dependence and trust as a privilege.

*That* [palliative care] *is a particularly emotional form of medicine ... you know, they chose me. That’s an amazing privilege.* [FG5-GP2].

Financial remuneration was not a major motivator for GPs in providing palliative care, although there was a sense of discouragement over the low rate of reimbursement and the long hours involved.

*I think* [for] *most people who work in palliative care, money is not their driving motivator … But, on the whole, I don’t know, financially, it isn’t worth it … It is a little about dedication.* [FG3-GP4].

General practitioners felt they altruistically provided support for their dying patients. However, one palliative care specialist reported that younger GPs hold a different view and were more likely to be influenced by financial benefits.

*Frankly, why would you make yourself available at the weekend if you are not making money? I think doctors over 50 have traditionally been brought up on that idea of providing service. I am not saying younger doctors don’t want to serve, just they tend to want to do it at their convenience.* [PCS3].

### Challenges and barriers in providing EOL care

Challenges and barriers were grouped into four major components: (i) Stewardship and structures of care; (ii) Educational development; (iii) Organisational processes, and (iv) Inter-personal processes.

#### Stewardship and structures of care

Participants widely voiced concerns that the Medicare Benefits Schedule payment model used in Australia tended to disadvantage GPs who adopted a holistic approach to the care of patients at EOL. The current scheme failed to compensate GPs for time spent travelling to home visits, phone calls, family meetings, advance care planning discussions, and organising case conferences. Inappropriate reimbursement for home visits had greater impact on rural GPs because of the longer travel time for home visits in sparsely populated places.

*… it’s actually time taken with the patient* [that is included] *in the Medicare descriptor* [for payment]*, not time getting there. We are extremely disadvantaged* [with regard to the Medicare Benefits Schedule in comparison to] *the people who may do the same sort of work in the areas where they live closer to the patient. So, to strictly apply the* [Medicare Benefits Schedule]*, you do yourself a disservice, and as a result, would discourage involvement in home care.* [FG2-GP1].

Participants highlighted limitations in human and material resources for provision of rural/regional EOL care. Limited access to medications after-hours, community-based palliative care services, social workers and hospital care were frequently reported. In addition, GPs’ limited awareness of local resources restrained effective use of the existing resources. This resulted in unnecessary hospital admissions, especially after-hours.

*… how many times, after hours on the weekend, do you do a ring around pharmacies from everywhere between here and [regional town named] chasing stuff down … I send patients up to [regional town cited] to pick stuff up...* [FG3-GP3].

Fewer transportation options in isolated places prevented patients from attending medical appointments and limited the number of home visits care providers could conduct. Furthermore, the poor living arrangements in remote places also raised GPs’ concerns around the safety of patients and medical teams doing home visits.

*There is no bus* [from the remote town]*, nothing. So,* [the patient may] *either miss the* [medical] *appointment or miss the bus …* [There is also a question over] *whether the* [home visit] *team can actually easily go there, and whether the place is actually appropriate to* [go to]*. I think* [that patient’s residence is] *not even a house, a shack we call it. The roof is probably going to fall or animals are going to come in or whatever. So the safety for the team going there is also a problem* [and] *that* [is] *something we need to think about.* [FG1-GP2].

It was perceived that any supporting service for GPs were more likely to make a difference in more isolated areas given the greater problems in accessing resources.

*The groups* [that] *often benefit from support are going to be the GPs who are working more remotely than* [regional town cited] *… If you are looking to provide support, services for rural practitioners are more likely to have an impact.* [ID-GP3].

Participants also recognised that the unavailability of care and resources in remote places sometimes made dying at home unrealistic, even though it was acknowledged that patients should be supported to die at home if they wish so, where possible.

*What about the question that patients want to die at home, but lack the support for that … The relatives cannot cope, syringe drivers aren’t available, there is a lack of nursing availability...* [FG3-GP7]

#### Educational development

Many of the training providers and GP recipients had a passion for palliative care education. However, GPs still considered training was insufficient because of a range of difficulties, especially in relation to obtaining funding to attend training programs that took them away from their work.

*I think the other thing is, it’s certainly our experience, when we offer the education, we are very keen, I think the doctors are very keen. … But one of problems is how they get funded to do it.* [PM4].

Insufficient training could also relate to the lack of a defined palliative care component in the medical curriculum and few opportunities for younger GP registrars to review EOL patients.

*It* [palliative care education] *is not really on the radar a great deal. It’s quite bound by the current curriculum. The students often come down* [to the rural clinical school] *for a year, they sort of have a broad range of experience, there is not a specific focus on palliative care*. [ID-GP2].

*When I, myself, go to talk to some GPs in training, or GP registrars, they often tell me that GP registrars can do a 6-month attachment in general practice, and they may not see one single patient that they think is in the last 12 months of life, because if patients are really unwell, they don’t tend to see the GP registrars who will be only in the practice for a short time.* [PCS2].

Time pressure was one of the major impediments for rural/regional GPs to attend palliative care training. They therefore preferred short case study-based education sessions by palliative care specialists in evenings or lunch breaks, as opposed to long-term training programs.

*The easiest* [way to provide palliative care training] *would be evening sessions. If it’s really involved a day or 2 days course, you probably wouldn’t get that level of interest to have such a long course. Other options are* [to] *go around and visit the practice, do lunch time visits.* [ID-GP1].

Although rural/regional GPs were perceived as having fewer training opportunities than urban GPs, some GPs reported that close relationships with community palliative care teams helped to improve their skills and confidence in providing palliative care.

*I think in [regional town cited], the GPs are particularly well-skilled in palliative care because of the palliative care team.* [There are] *yearly conferences, workshops, and [the local palliative care specialist] is very available, the nurses are very knowledgeable.* [FG3-GP3].

#### Organisational processes

All participants recognised that continuity of care was reduced by the incomplete and fragmented information transfer between GPs and hospitals. Patients’ discharge summaries were often shared too late and contained inadequate information.

*The biggest problem is information transfer. For example, the hospital discharge summary can be slow and sometimes don’t come through in a timely way.* [PCS3].

Conversely, information-sharing with local, often nursing-based, palliative care teams was timely and collegial, and helped to promote holistic and collaborative multidisciplinary care.

*I think our palliative care service, especially the nursing staff, is fantastic, very responsive, and very keen to see patients at home and feedback to us. I always get a follow-up email after they have been seeing anyone who I referred to them.* [FG1-GP2].

Although GPs considered that case conferences were a good idea in principle, they found organising them time-consuming and therefore sought clear pre-established procedures and a designated coordinator (ideally a practice nurse).

*For me, to set up a multi-disciplinary meeting, I have to research the process first—who is going to be involved, engaging with all of the particular players involved—then put all together.* [Then] *I don’t have the time to actually do the homework* [before the meeting].*..* [FG2-GP2].

A number of practitioners noted the importance of their relationships with the community palliative care team and ease of accessing palliative care support. One GP, however, reported feelings of guilt about referring patients to specialist palliative care, viewing referral as “abandoning” patients. Palliative care specialists described behaviours that limited collaboration with some GPs, such as referrals that were too late to be of maximum benefit, GPs’ being unsure about accessing palliative care specialists, and GPs not giving consent for palliative care specialists to review their patients. Palliative care specialists suggested that unclear role delineation contributed to these concerns and identified the need for greater integration of palliative care into primary care.

*That comes down to professional and personal situations, like, who* [is now primarily responsible for] *the patient? Is the team going to take over? Are they telling me what to do …*? [PCS1].

Most participants felt that rural areas had better care integration and continuity than urban areas. For example, rural GPs highlighted the advantages of GP-run hospitals/hospices and on-call rosters in maintaining the continuity of their care, as well as the support from the state-based palliative care specialist telephone helpline.

*I do think our access to hospice is very good here [in a regional town] …, so for respite, even though they plan to die at home, having access to the hospice has been very very good. … If you say the carers just need a break,* [they can] *get the patients into hospice.* [FG2-GP3].

#### Inter-personal dynamics and processes

Undertaking conversations about disease progression, deterioration, goals of care and advance care planning with patients and their carers were viewed as essential components of EOL care but were often considered challenging. General practitioners and other stakeholders identified concerns about a range of issues: patients’ strong emotional reactions to bad news, particularly in younger patients; difficulties in answering patients’ questions due to insufficient, delayed or unclear information from other professionals; unrealistic requests from patients and families and family conflicts about care; cultural differences between GPs and patients; and GPs’ time limitations. Because of relatively limited access to palliative care physicians, rural GPs had to confront these challenges themselves more frequently than urban GPs.

*One of the more challenging parts of the tasks for GPs, sometime it’s family negotiation, very unrealistic expectation from family. They fly in from wherever to spend their last days, wondering why there isn’t IV* [intravenous therapy] *fluid running, or why we aren’t doing a feeding tube or something... It can be very challenging part. And it often falls at our feet. I am sure there are palliative care physicians that deal with that at metro areas hospices.* [FG4-GP3].

However, some GPs in small towns perceived that the closer healing relationships made EOL discussions easier.

*Everyone knows each other, arguably everyone’s related, there is a bit of a tight community feel. I think palliative care in [small town cited] is a lot easier.* [FG5-GF2].

## Discussion

This study presented multidisciplinary perspectives on the involvement of rural/regional GPs in provision of palliative and EOL care and the complex challenges they face. Resource limitations and lack of training frustrated GP attempts to provide comprehensive EOL care. Burdensome payment models contributed to a reluctance to engage with some aspects of EOL care such as case conferences, advance care planning discussions, and home visits.

A number of these findings on the perceived role of GPs in providing EOL care and barriers they experience are comparable to those of previous studies [18, 24, 36–39], including studies that have focused on urban GPs [40]. However, the current study indicated that many of these barriers appear to be more pronounced in rural versus urban contexts within Australia. For example, under the current Medicare payment system, the lack of recognition of time spent travelling to home visits disproportionately disadvantages rural GPs because they must travel longer distances. Remoteness also contributes to less availability of clinical resources and options at EOL [9, 24, 41], thereby compromising patients’ wishes to die at home and increasing the burden of care provision on rural GPs.

There was evidence that the study participants perceived closer GP-patient relationships and better care integration and collaboration in rural versus urban areas. These factors may help to facilitate EOL conversations and improve the continuity of GPs’ care, as well as their confidence and skills in palliative care delivery. These are important advantages for rural GPs given that interactions with other professionals and patients are reportedly viewed as more challenging than the basic “technical” aspects of palliative care. The responses from focus groups and interviews also suggest that rural GPs often take more direct responsibility for patient EOL care than their urban counterparts [16, 17]. This greater degree of direct professional responsibility - coupled with suboptimal resource availability - emphasises the importance of strengthening rural primary care and making better use of existing resources, particularly given the accelerating demands for palliative care service in Australia’s ageing regional communities [14]. Also, GPs’ limited awareness of local resources constrained their effective utilisation and confirmed the need for better organization of resources in rural areas. An information guide identifying local available resources could be helpful [42].

Previous studies have found that GPs feel guilty about reporting financial benefits as a motivator for providing EOL care [43]. General Practitioners in this study did not consider financial benefits as a factor in care, despite expressing criticism over the current fee-for-service payment model. Our findings are in line with the growing calls for the development of Medicare items specifically for GP palliative care and for the simplification of claim processes [44] to ensure financially feasible and sustainable community-based systems [14, 45]. Duckett has noted the lower incentives for community-based palliative care compared to hospital-based palliative care in Australia and recommends an activity-based bundled funding model which incorporates performance monitoring and reporting [46]. To facilitate such reforms, we require a better understanding of rural patients’ needs for palliative care and GPs’ activities in providing this care.

Our study indicated insufficient palliative care training at both undergraduate [47] and postgraduate [37–39] levels in rural areas. All clinicians who are involved in primary palliative care should have core skills and competencies in the field [48]. Recent surveys showed around 30% of Australian GPs had lower confidence and interest in providing palliative care because of inadequate training, despite the majority of them stating that they wanted to learn more [49, 50]. Governments are increasingly being called upon to invest more in palliative care training in primary care [51]. In rural areas, busy schedules and long distances may have hindered GPs’ access to palliative care training. Distance learning programs [52, 53] and palliative care specialist practice visits could help address these barriers by offering a flexible study schedule and eliminating geographical barriers. However, a number of GPs in this study were not aware of the available training opportunities. More effective channels are needed to inform rural GPs of these options. More generally, younger GPs should be encouraged to provide EOL care to maintain their role and gain experience in the context of increasing specialisation of this medical discipline.

Widespread problems in information transfer, case conferences and late palliative care referrals reported in this study reflect a degree of fragmentation of the healthcare system, and have undermined coordination and continuity of care at EOL [54]. Globally, there are increasing calls for reforms that would allow government health agencies to integrate palliative care into primary care [51, 55, 56]. However, it is still unclear how such integration would be achieved and the process is likely to be challenging [57–59]. Lack of both standardised clinical pathways and systems for information-sharing have impeded the establishment of stable and integrated EOL care across relevant agencies [60]. Efforts to develop web-based system to monitor and refer patients have encountered multiple difficulties because of concerns about data security [61] and heterogeneity in illness trajectories [62, 63].

To avoid overwhelming the already limited healthcare resources, involvement and support from both internal and external players are needed. Internal players (i.e. primary care and palliative care providers, local healthcare organisations) should ensure local needs and capacity are fully considered, whereas external players need to strengthen healthcare infrastructures through evidence-based decisions on funding, staffing, regulation and legislation [58, 60, 64].

### Strengths and limitations

To our knowledge, this is the first research project to provide a fully integrated picture of rural GPs’ involvement and challenges in providing palliative and EOL care in Australia. A more comprehensive understanding of the challenges in providing this care was provided by our involvement of study participants with different cultural perspectives, professional backgrounds and roles within the wider health system. However, this study had a number of limitations including time constraints and at times disruptive settings for conducting focus groups in GPs’ rooms. However, in all circumstances, we did ensure that all participating GPs answered the core questions relating to their EOL care.

## Conclusions

In their provision of EOL care, rural GPs share a number of common challenges with urban GPs but appear to be disproportionately affected by inappropriate payment models and limited resources. There is evidence that rural GPs enjoy closer doctor-patient relationships and better care integration and collaboration than those in urban areas. However, it is still unclear how to build on these strengths and address the challenges without a more complete understanding of how and what palliative care is provided for EOL patients in rural Australia. A system for data collection on the management of individual patients would help to provide empirical data and clarify the scope of rural GPs’ care at the EOL.

## Additional file

Additional file 1: Interview guide questions. (DOCX 24 kb)

## Data Availability

The datasets supporting the conclusions of this article are available from the corresponding author upon request.

## Abbreviations

EOL: End-of-life
FG: Focus group
GPs: General practitioners
ID: In-depth interview
PCR: Palliative care researcher
PCS: Palliative care specialist
PM: Policy maker
WA: Western Australia
