# Supplementary material for: End-of-life care in rural general practice: how best to support commitment and meet challenges?
Source: BMC Palliat Care. 2019 Jun 25;18:51. doi: 10.1186/s12904-019-0435-4 (PMC6593492; doi:10.1186/s12904-019-0435-4)
Supplement: Supplementary file 1 — Interview guide questions. (DOCX 24 kb) [file 12904_2019_435_MOESM1_ESM.docx]

**Guide questions for GPs**

Please now think about the last two patients you cared for who died with an advanced chronic disease.

1. What do you consider your role was in providing care for these patients in their last 12 months of life?
2. What other services did you use to help care for these two patients in their last 12 months of life?
3. How would you describe your experience of collaboration with these services?
4. How did you communicate with these two patients about their condition and prospects of death in the near future?

1. How were decisions made concerning the patients’ end of life care?
2. What factors do you think had positively impacted your care delivery to these two patients?
3. What factors do you consider had produced negative impact on your care delivery to these two patients?
4. In your opinion, how could the care for these two patients in the last 12 months of life be improved?

**Guide questions for other stakeholders**

1. How would you describe your professional role in managing or providing palliative and end of life care?
2. What do you consider the GPs’ role is in providing care for patients in their last 12 months of life?
3. Can you describe how you work in conjunction with GPs in providing end of life care?
4. How do you think this partnership might be improved?
5. What do you consider to be the main difficulties and challenges for rural GPs in providing palliative and end of life care in community setting?
6. What do you consider to be the main advantages or facilitators for rural GPs in providing palliative and end of life care in community setting?
7. How can end of life care be supported/improved in general practice?
8. In your opinion, how could the care for patients in the last 12 months of life be improved?
